# The complete chloroplast genome of *Bauhinia racemosa* Lam. (Fabaceae): a versatile tropical medicinal plant

**DOI:** 10.1080/23802359.2022.2110010

**Published:** 2022-08-22

**Authors:** Yan Xiao, Ya-Ya Qu, Chun-Hui Hao, Lu Tang, Jiao-Lin Zhang

**Affiliations:** a Xishuangbanna Tropical Botanical Garden, Chinese Academy of Sciences, Mengla, China; b University of Chinese Academy of Sciences, Beijing, China; c Millennium Seed Bank, Royal Botanic Gardens Kew, Wakehurst, West Sussex, UK; d State Forestry and Grassland Administration, Southwest Forestry University, Kunming, China

**Keywords:** *Bauhinia racemosa*, Cercidoideae, chloroplast genome, Fabaceae, phylogeny, plastome

## Abstract

*Bauhinia racemosa* Lam. (1783), a versatile medicinal plant, belongs to the family Fabaceae (subfamily Cercidoideae). In this study, we analyzed the complete chloroplast genome to facilitate its use in genetic research. The complete chloroplast genome of *B*. *racemosa* was found to be 155,501 bp long, including two inverted repeat (IR) regions of 25,446 bp, which are separated by a small single-copy (SSC) region of 18,295 bp and a large single-copy (LSC) region of 86,314 bp. The overall GC content is 36.4%. The genome of *B*. *racemosa* contains 129 genes, including 83 protein-coding genes, 37 tRNAs, 8 rRNAs, and 1 pseudogene (rps19). Phylogenetic analysis suggests that *B*. *racemosa* forms a monophyletic clade with the other four *Bauhinia* species (*B. brachycarpa*, *B. purpurea*, *B. blakeana* and *B. variegata* var. *variegata*).

*Bauhinia racemosa* Lam., commonly known as the bidi leaf tree, is a small (up to 12 metres) deciduous species of tree with dark scabrous bark and is widely distributed in tropical areas of South, Southeast and East Asia, which are characterized by harsh climatic conditions (Panda et al. 2015). The tree is important nutritionally and economically, with its leaves serving as fodder for livestock and its hard and heavy wood being used as fuel (Panda et al. 2015). More significantly, *B. racemosa* is also used in traditional medicine with almost every part of the plant having some medicinal value. The flower buds have anti-ulcerogenic properties (Akhtar and Ahmad 1995), the seeds can be exploited for their antibacterial benefits (Kumar et al. 2005), and the isolated compounds from the roots exhibit profound antibacterial and antifungal activity (Jain et al. 2008). In addition, its leaf extracts have antihyperglycemic and anthelmintic properties (Prusty et al. 2012), and its stem bark is reported to be medicinally important for treating a range of ailments, e.g. headache, fever, skin diseases, and diarrhea (Borikar et al. 2009). Although the plant is known to be important for human use, there has only been a limited number of genomic studies on this species.

In this analysis, young leaves of *B. racemosa* were collected from Xishuangbanna Tropical Botanical Garden, Chinese Academy of Sciences, Yunnan Province, China (XTBG, 21°41′N, 101°25′E). A voucher specimen, reference number F2020012, was deposited at the Herbarium of Xishuangbanna Tropical Botanical Garden (HITBC) (http://hitbc.xtbg.ac.cn, Jianwu Li, ljw@xtbg.org.cn). DNA sequencing was performed by the Personal Biotechnology Co., Ltd (Shanghai, China), where the CTAB method (Doyle 1987) was used to extract the total genomic DNA of leaves, and the Illumina NovaSeq 6000 sequencing platform was used to generate 2 × 150 bp paired-end reads. A total of 3.46 G bases of raw data were trimmed and filtered by Fastp software (Chen et al. 2018). The chloroplast genome of *B*. *racemosa* was assembled and annotated using default parameters of the GetOrganelle toolkit (Jin et al. 2020) and the web server CPGAVAS2 (Shi et al. 2019), respectively. In addition, Geneious v.8.1.3 software (Kearse et al. 2012) was used to check and correct erroneous gene names after an auto-annotation. The annotated sequence was submitted to Genbank with the accession number ON456405.

The annotation results show that the complete chloroplast genome of *B*. *racemosa* is a circular DNA molecule with a length of 155,501 bp, which is 47 bp shorter than *B*. *brachycarpa* (NC037762). The plastome of *B. racemosa* contains a small single-copy region (SSC) of 18,295 bp, a large single-copy region (LSC) of 86,314 bp, and two inverted repeat (IR) regions of 25,446 bp. The overall GC content is 36.4%. The GC content is the highest in IR regions (42.5%), the corresponding values of the SSC and LSC are 30.5% and 34.1%, respectively. The complete chloroplast genome encoded 129 genes, including 83 protein-coding genes, 37 tRNA genes, 8 rRNA genes, and 1 pseudogene (rps19).

To determine the phylogenetic position of *B*. *racemosa*, the complete chloroplast genomes of 16 additional Fabaceae species from the subfamily Cercidoideae were downloaded from GenBank and all protein coding gene sequences were compared using the MAFFT alignment method by Geneious v.8.1.3 software (Kearse et al. 2012). A maximum-likelihood analysis was performed with IQ-TREE v.1.6.7 (Nguyen et al. 2015) with the best-fit model TVM + F+R2 automatically selected by ModelFinder (Kalyaanamoorthy et al. 2017). *Cercis canadensis* (KF856619) and *C. glabra* (NC036762) were selected as the outgroups. Phylogenetic analysis suggests that *B*. *racemosa* forms a monophyletic clade with the other four *Bauhinia* species (*B. brachycarpa*, *B. purpurea*, *B. blakeana* and *B. variegata* var. *variegata*) (Figure 1). The basic structure of our phylogenetic tree was consistent with that seen in a previous study (Gu et al. 2020).

**Figure 1.**
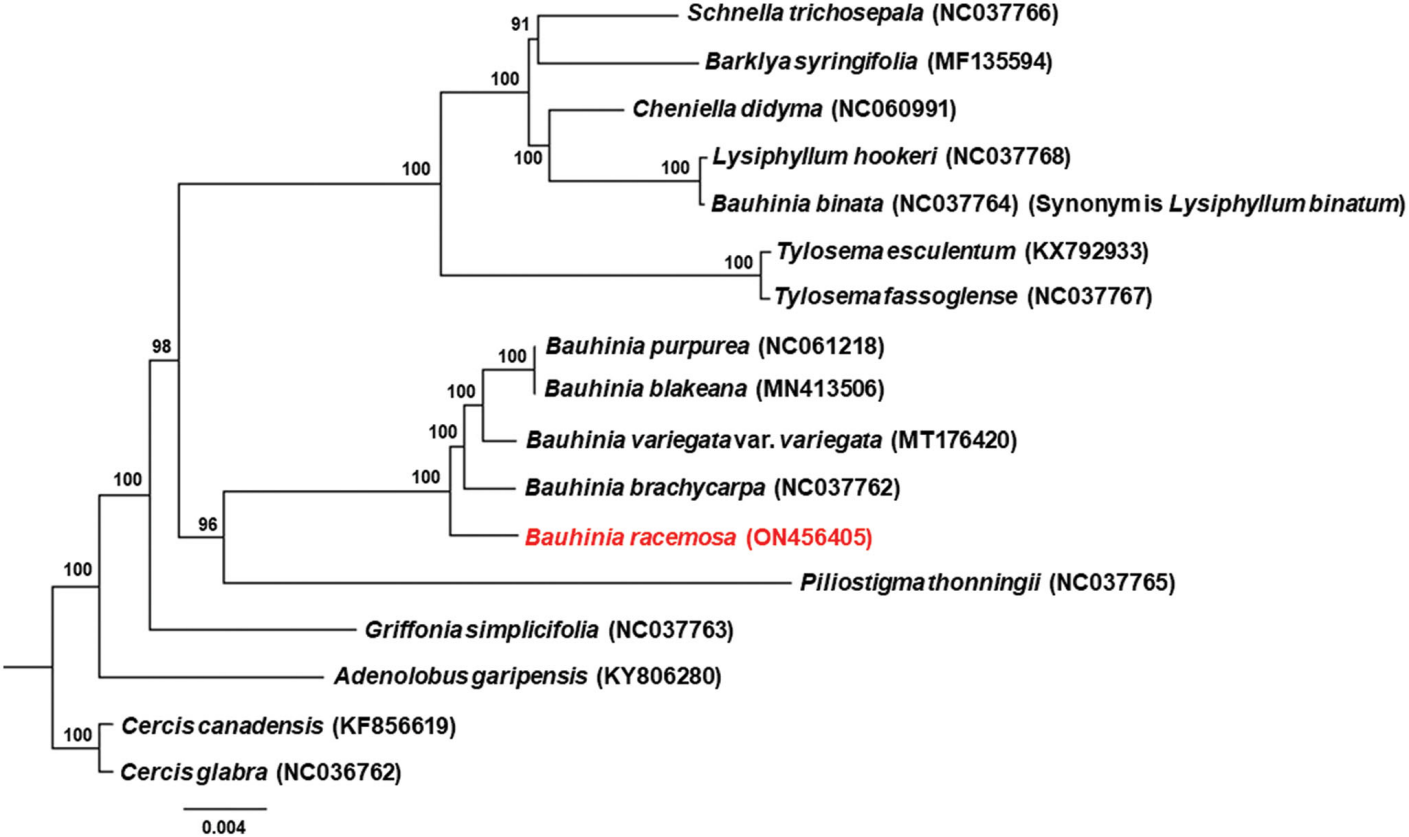
The maximum-likelihood phylogenetic tree for *B. racemosa* based on 71 protein-coding genes from the plastomes of 16 other Cercidoideae species. The outgroup species are *Cercis canadensis* and *C*. *glabra.*

## Data Availability

The genome sequence data that support the finding of this study are openly available in GenBank of NCBI at (https://www.ncbi.nlm.nih.gov) under the accession no. ON456405. The associated BioProject, SRA, and Bio-Sample number are PRJNA843449, SRS13209563, and SAMN28742914, respectively (https://www.ncbi.nlm.nih.gov/bioproject/PRJNA843449).

## References

[CIT0001] Akhtar AH, Ahmad KU. 1995. Anti-Ulcerogenic evaluation of the methanolic extracts of some indigenous medicinal plants of Pakistan in aspirin ulcerated rats. J Ethnopharmacol. 46(1):1–6. 7475118 10.1016/0378-8741(94)01220-t

[CIT0002] Borikar VI, Jangde CR, Philip P. 2009. Study of antipyretic activity of *Bauhinia racemosa* Lam in rats. Vet World. 2(6):217–218.

[CIT0003] Chen S, Zhou Y, Chen Y, Gu J. 2018. Fastp: an ultra-fast all-in-one FASTQ preprocessor. Bioinformatics. 34(17):i884–i890. 30423086 10.1093/bioinformatics/bty560 PMC6129281

[CIT0004] Doyle JJ. 1987. A rapid DNA isolation procedure for small quantities of fresh leaf tissue. Phytochem. Bull. 19(1):11–15.

[CIT0005] Gu SR, Chen Y, Zheng DJ, Meng SY, Tu TY. 2020. The complete plastid genome of *Bauhinia variegata* L. var. *variegata* (Leguminosae). Mitochondrial DNA B. 5(2):1701–1702.

[CIT0006] Jain R, Saxena U, Rathore K, Jain SC. 2008. Bioactivities of polyphenolics from the roots of *Bauhinia racemosa*. Arch Pharm Res. 31(12):1525–1529. 19099218 10.1007/s12272-001-2145-7

[CIT0007] Jin JJ, Yu WB, Yang JB, Song Y, dePamphilis CW, Yi TS, Li DZ. 2020. GetOrganelle: a fast and versatile toolkit for accurate de novo assembly of organelle genomes. Genome Biol. 21(1):241. 32912315 10.1186/s13059-020-02154-5 PMC7488116

[CIT0008] Kalyaanamoorthy S, Minh BQ, Wong TKF, von Haeseler A, Jermiin LS. 2017. Model finder: fast model selection for accurate phylogenetic estimates. Nat Methods. 14(6):587–589. 28481363 10.1038/nmeth.4285 PMC5453245

[CIT0009] Kearse M, Moir R, Wilson A, Stones-Havas S, Cheung M, Sturrock S, Buxton S, Cooper A, Markowitz S, Duran C, et al. 2012. Geneious basic: an integrated and extendable desktop software platform for the organization and analysis of sequence data. Bioinformatics. 28(12):1647–1649. 22543367 10.1093/bioinformatics/bts199 PMC3371832

[CIT0010] Kumar RS, Sivakumar T, Sunderam RS, Gupta M, Mazumdar UK, Gomathi P, Rajeshwar Y, Saravanan S, Kumar MS, Murugesh K, et al. 2005. Antioxidant and antimicrobial activities of *Bauhinia racemosa* L. stem bark. Braz J Med Biol Res. 38(7):1015–1024. 16007272 10.1590/s0100-879x2005000700004

[CIT0011] Nguyen L-T, Schmidt HA, von Haeseler A, Minh BQ. 2015. IQ-TREE: a fast and effective stochastic algorithm for estimating maximum-likelihood phylogenies. Mol Biol Evol. 32(1):268–274. 25371430 10.1093/molbev/msu300 PMC4271533

[CIT0012] Panda P, Debajyoti D, Priyanka D, Goutam G. 2015. Therapeutic potential of *Bauhinia racemosa* – a mini review. Int J Pharm Sci Rev Res. 32(2):169–179.

[CIT0013] Prusty KB, Rao JV, Subudhi SK, Reddy PA, Raj KJ. 2012. Anti hyperglycemic activity of extracts of leaves of *Bauhinia racemosa* Lamk (Family-Caesalpineaceae) on normal and alloxan-induced diabetic rats. IJPRAS. 1(4):94–99.

[CIT0014] Shi LC, Chen HM, Jiang M, Wang LQ, Wu X, Huang LF, Liu C. 2019. CPGAVAS2, an integrated plastome sequence annotator and analyzer. Nucleic Acids Res. 47(W1):W65–W73. 31066451 10.1093/nar/gkz345 PMC6602467

